# Assessing the Economic and Environmental Viability of *Undaria pinnatifida* Sporophylls as Sustainable Cattle Feed in South Korea

**DOI:** 10.3390/ani15223260

**Published:** 2025-11-10

**Authors:** Hyeseong Kim, Pilgyu Jung, Yonghyun Do, Jungjun Park, Sam-Churl Kim, Bong-Tae Kim

**Affiliations:** 1 Aquaculture Research Division, National Institute of Fisheries Science, Busan 46083, Republic of Korea; hyekim@korea.kr (H.K.); hyun5719@korea.kr (Y.D.); pjj515@korea.kr (J.P.); 2 Resource & Environmental Economics Research Institute, Pukyong National University, Busan 48548, Republic of Korea; moonshine91@naver.com; 3 Division of Applied Life Science (BK21Four, Institute of Agriculture and Life Science), Gyeongsang National University, Jinju 52828, Republic of Korea; kimsc@gnu.ac.kr; 4 Division of Marine & Fisheries Business and Economics, College of Fisheries Science, Pukyong National University, Busan 48513, Republic of Korea

**Keywords:** seaweed by-product, *Undaria pinnatifida* sporophylls, Hanwoo cattle, methane abatement, economic feasibility, sustainable aquaculture, livestock decarbonization

## Abstract

This study analyzed the economic feasibility of utilizing *Undaria pinnatifida* sporophylls (UPSs), a major by-product of seaweed aquaculture, as feed for Hanwoo cattle. It employed a partial equilibrium framework to quantify processing costs, avoided disposal costs, substitution savings from reduced grain imports, and monetized methane abatement benefits, calibrated with national statistics on Hanwoo production and feed use for 2022–2024. The analysis revealed that, in the absence of environmental valuation, additional collection, transport, and drying costs (KRW 25,714–102,857 per head at 0.25–1.0% inclusion) outweighed savings from disposal avoidance and import substitution. When methane abatement was priced under Korea’s Emissions Trading Scheme, however, net benefits emerged, ranging from KRW 22,757 to 40,859 per head, with welfare gains of KRW 19,108 million at 1.0%. Sensitivity analysis confirmed a strong dependence on carbon prices: benefits were substantial at KRW 40,000 per ton of CO_2_, remained positive yet relatively limited at KRW 20,000, and shifted into negative territory at KRW 10,000. These findings demonstrate that UPS feed utilization can advance climate mitigation and feed security, provided it is supported by effective carbon pricing and producer-oriented policies.

## Simple Summary

Seaweed aquaculture generates substantial amounts of by-products that are often discarded, creating both waste and environmental challenges. In Korea, the sporophylls of sea mustard (*Undaria pinnatifida*) represent a major by-product of seaweed aquaculture. Rather than being treated as waste, these by-products can be recycled as feed for Hanwoo cattle (*Bos taurus coreanae*), thereby promoting circular resource use and strengthening feed security. This study evaluated the economic and environmental effects of such recycling by considering processing costs, savings from avoided disposal and reduced grain imports, and the benefits of methane abatement. The analysis showed that, without accounting for environmental benefits, costs exceeded savings. However, when methane abatement was valued according to Korea’s carbon trading system, net benefits emerged, particularly at higher inclusion levels, but only when carbon prices were sufficiently high. These results suggest that seaweed by-products can contribute simultaneously to climate mitigation and feed resilience. Policy instruments such as carbon pricing and targeted subsidies will be essential to ensure long-term viability for producers.

## 1. Introduction

Seaweed aquaculture has expanded rapidly over recent decades, emerging as one of the most dynamic sectors in global marine production. This growth has been fueled by rising demand for sustainable marine resources with applications in food, animal feed, pharmaceuticals, and biotechnology [1,2,3,4].

This expansion has relied heavily on a few key taxa, notably laver (*Porphyra*), sea mustard (*Undaria pinnatifida*), and kelp (*Saccharina japonica*), which together account for a substantial share of global output. South Korea is a leading producer of these species, with their combined average annual output reaching approximately 2.27 million tons from 2022 to 2024. This output represents 76% of the nation’s marine aquaculture production by wet weight [5].

Despite significant growth, the seaweed industry continues to face structural challenges, particularly the underutilization of by-products and the costs of their disposal. Improper practices such as illegal landfilling and ocean dumping create environmental externalities and broader societal burdens [6,7,8]. Evidence from the Caribbean illustrates the scale of these impacts: Rodríguez-Martínez et al. [9] estimate annual *Sargassum* removal costs of USD 300,000–1.1 million per kilometer of coastline, while Schuhmann et al. [10] document substantial economic losses affecting tourism, fisheries, and coastal property. Although these cases involve wild *Sargassum* rather than aquaculture by-products, they demonstrate that unmanaged biomass can disrupt ecosystem services and impose considerable costs. In Korea, sea mustard (*Undaria pinnatifida*) represents a critical case, given its large-scale production and high by-product generation. This situation underscores the need for scalable, sustainable utilization strategies.

Against this backdrop, scholarly attention has increasingly turned to the valorization of seaweed by-products as a means of transforming an environmental burden into an economic opportunity. This study is motivated by Korea’s heavy reliance on imported feed, the rising costs of seaweed disposal, and the potential of *Undaria pinnatifida* sporophylls (UPSs) to reduce methane emissions in cattle production. One promising avenue lies in their use as alternative feed resources for ruminants. Numerous studies have demonstrated the potential of seaweed by-products, particularly those derived from *U. pinnatifida*, as alternative feed ingredients for ruminants. Feeding trials have shown that the inclusion of *U. pinnatifida* by-products in dairy cow diets can stabilize rumen pH, increase milk yield, and enhance calcium content [11,12]. Other studies report that partial substitution of soybean meals with algal by-products does not adversely affect digestibility [13,14], suggesting that the protein-rich composition of seaweeds could reduce dependence on conventional protein feeds. Moreover, seaweed extracts have been found to lower enteric methane emissions while improving feed efficiency, thereby providing both nutritional and environmental benefits [15,16,17,18].

Considering these developments, the National Institute of Fisheries Science (NIFS) in Korea initiated research in 2023 on the potential use of sea mustard by-products in cattle feed. According to the NIFS [19], methane reduction rates observed in cattle feed trials were 0% for kelp (*S. japonica*), 16% for laver (*Porphyra spp*.), 9–17% for sea mustard (*U. pinnatifida*), and as high as 23% for its sporophylls (UPSs), indicating the comparatively greater mitigation potential of UPSs. On this basis, the present study focuses on UPSs, which have exhibited particularly strong methane-mitigation effects in experimental settings [19,20]. While these findings underscore the biological potential of UPSs, the economic dimension has only recently been addressed. Kim et al. [21] provided the first economic evaluation of incorporating aquaculture seaweed by-products into cattle feed in Korea and concluded that feed substitution and methane abatement could yield considerable benefits. However, their analysis was constrained by the restrictive assumption that market equilibrium would remain unchanged, thereby neglecting possible adjustments in equilibrium resulting from shifts in production costs. The present study addresses this limitation by examining the economic feasibility of UPS utilization under more realistic conditions in which supply responses are endogenously adjusted to reflect changes in production costs.

Against this background, the present study conducts a comprehensive economic assessment of UPS utilization by jointly considering costs and benefits. On the cost side, additional expenditures arise from collection, transport, and feed processing. On the benefit side, UPS utilization can reduce dependence on imported feed ingredients, thereby enhancing domestic feed security, while also lowering the social costs associated with by-product disposal and generating environmental gains through greenhouse gas abatement. Specifically, the objectives of this study are to (1) estimate the incremental costs and cost savings associated with UPS utilization, (2) quantify methane abatement benefits and their monetized value under alternative carbon price scenarios, and (3) evaluate the overall effects on consumer surplus, producer surplus, and social welfare. By pursuing these objectives, this study provides a rigorous assessment of the economic feasibility of UPSs as a feed resource and identifies policy implications for the sustainable management of seaweed by-products.

## 2. Materials and Methods

### 2.1. Economic Effect of Using UPSs as Feed Ingredients

**Figure 1:**
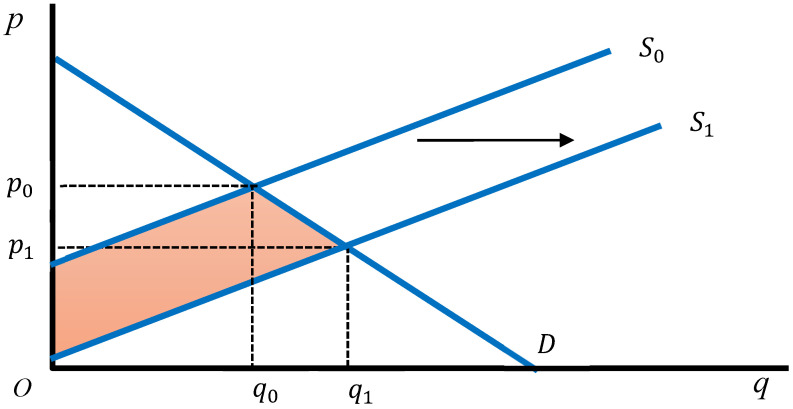
Welfare effects of a rightward shift in the beef supply curve. The colored area indicates the increase in social welfare.

The economic effects of incorporating UPSs into cattle feed could be evaluated by measuring changes in consumer surplus and producer surplus in the beef market, where beef is the final output. Adjustments in feed inputs affect production costs and productivity, which are transmitted as shifts in the supply curve. A rightward shift in the supply curve, arising from cost reductions or productivity improvements, results in a lower equilibrium price and higher equilibrium quantity. Under these conditions, both consumer and producer surplus alter, and the aggregate social surplus, defined as their sum, increases by the shaded area shown in Figure 1. The shifts in the supply curve are assumed to be parallel, reflecting the assumption that cost reductions from UPS utilization and the associated decrease in greenhouse gases, particularly methane, are linearly related to beef output.

In this framework, the parameter k is defined as the proportionate reduction in production costs attributable to UPS utilization. Following the formulation of Alston et al. [22], the resulting changes in consumer surplus (*ΔCS*), producer surplus (*ΔPS*), and total social surplus (*ΔTS*) are expressed as functions of the initial equilibrium price (*p*_0_), quantity (*q*_0_), demand elasticity (*η*), and supply elasticity (*ε*), and are given as follows:

(1) $$\Delta CS=-p_{0}q_{0}k\frac{\varepsilon }{\eta \left(\eta +\varepsilon \right)}$$

(2) $$\Delta PS=p_{0}q_{0}\;k\frac{\varepsilon }{\left(\eta +\varepsilon \right)}$$

(3) ΔTS=ΔCS+ΔPS

For the empirical application, demand and supply elasticities are assumed to be –1.33 and 0.64, respectively, based on estimates from the Korea Rural Economic Institute [23]. Previous studies suggest that demand elasticities generally range from –1.06 to –0.67, while supply elasticities generally range between 0.49 and 1.11 [23]. The most recent estimates are employed in this analysis, so the most recent estimates are therefore employed. Furthermore, consistent with Alston et al. [22], this study considered that welfare outcomes are not highly sensitive to variations in elasticity parameters, and that the application of alternative values yields broadly comparable results.

The quantity variable (*q*_0_) is calibrated using the number of Hanwoo cattle (*Bos taurus coreanae*) to ensure consistency with available statistical data. On this basis, the parameter *k* is estimated as a composite measure that integrates both additional expenditures and offsetting benefits. It encompasses new costs arising from the collection, transport, and processing of UPSs, as well as avoided costs of by-product disposal, the savings from substituting imported feed ingredients, and the monetized benefits of reduced greenhouse gas emissions, particularly methane. All monetary values are standardized on a per-head basis for Hanwoo cattle.

### 2.2. Estimation of Incremental Processing Costs

The incremental costs represent the additional expenditures incurred in processing UPSs into cattle feed. In the absence of cost data specific to UPSs, proxy values were adopted from food waste treatment estimates reported by the Ministry of Environment of Korea [24]. This proxy was deemed appropriate based on aquaculture by-products of *U. pinnatifida* in Korea generally being discarded at sea, whereas processing stage by-products are discharged with food waste streams [21]. Given that seaweed farming in Korea is geographically concentrated in a limited number of coastal regions, and the assumption that feed processing would likely occur locally, current freight rates are considered broadly representative of transport costs. Moreover, since UPS-based feed processing has not yet been commercialized, precise estimates of dry feed conversion costs are not available, necessitating reliance on standardized food waste feed-processing values as proxies.

These proxy values, originally reported in 2012, were subsequently updated to 2022–2024 price levels by applying the cumulative consumer price inflation rate of 21.1% [25], thereby ensuring the consistency of the cost basis. On this basis, the collection and transport of by-products were valued at KRW 79,247 per ton (outsourced vehicle transportation), and the cost of producing dried feed was estimated at KRW 87,868 per ton.

As these estimates are expressed on a wet-weight basis, they must first be converted into per-head equivalents to ensure analytical consistency. The average fattening period of Hanwoo steers from 2022 to 2024 was 31.1 months [26], and compound feed consumption over this period was approximately 6.155 tons per head [27]. Following the experimental framework of the National Institute of Fisheries Science [20], supplementation rations of 0.25%, 0.5%, and 1.0% were adopted, corresponding to 15.4, 30.8, and 61.5kg of UPSs (dry weight) per head, respectively. Given that the dry matter content of seaweed by-products is approximately 10% of wet weight [1], the raw material requirements are tenfold higher when expressed on a wet-weight basis. Accordingly, the feed-processing cost per head is expressed as:

(4) $$\mathrm{Feed}\mathrm{processing}\mathrm{cost}\left(\frac{\mathrm{KRW}}{\mathrm{head}}\right)=\{79,247\left(\frac{\mathrm{KRW}}{\mathrm{ton}}\right)+87,868\left(\frac{\mathrm{KRW}}{\mathrm{ton}}\right)\}\times 10\times 6.155\left(\frac{\mathrm{ton}}{\mathrm{head}}\right)\times \mathrm{Supplementation}\mathrm{ratio}$$

### 2.3. Estimation of Avoided Disposal and Substitution Costs

To assess the economic implications of UPS utilization, avoided and substitution costs are defined as the savings realized when UPSs are diverted from disposal for use as cattle feed. In Korea, aquaculture by-products are generally discarded at sea, and the Ministry of Oceans and Fisheries [28] has estimated disposal costs at KRW 60,000 per ton (wet weight). This expenditure represents a fiscal burden on the public sector, which could be reduced through the incorporation of UPSs into feed. Reliable national-level estimates for the cleanup or removal of UPSs are not currently available in Korea. Accordingly, the disposal cost for marine fishery by-products reported by the Ministry of Oceans and Fisheries [28] was adopted as a proxy value, providing a conservative basis for estimating avoided disposal benefits. Such avoided costs could, in principle, be transferred to producers through policy mechanisms such as direct payments of feed subsidies. On a per-head basis, the avoided disposal cost could be expressed as follows:

(5) $$\mathrm{Avoided}\mathrm{disposal}\mathrm{cost}\left(\frac{\mathrm{KRW}}{\mathrm{head}}\right)=60,000\left(\frac{\mathrm{KRW}}{\mathrm{ton}}\right)\times 10\times 6.155\left(\frac{\mathrm{ton}}{\mathrm{head}}\right)\times \mathrm{Supplementation}\mathrm{ratio}$$

Additionally, substantial savings arise from the substitution of imported feed ingredients. Korea is heavily dependent on imported grains, with approximately 79% of feed demand met through imports [29]. The quantity of incorporated UPSs (dry weight) can therefore be valued using the average import price of corn, wheat, and soybean meal from 2022 to 2024, which is KRW 444,433 per ton [30,31,32]. On a per-head basis, the substitution savings are calculated as:

(6) $$\mathrm{Substitution}\mathrm{savings}\left(\frac{\mathrm{KRW}}{\mathrm{head}}\right)=444,433\left(\frac{\mathrm{KRW}}{\mathrm{ton}}\right)\times 6.155\left(\frac{\mathrm{ton}}{\mathrm{head}}\right)\times \mathrm{Supplementation}\mathrm{ratio}$$

### 2.4. Estimation of Methane Abatement Benefits

The environmental benefits of UPS utilization were evaluated through the monetization of methane emission reductions from enteric fermentation. The reduction rate was derived from in vitro rumen fermentation experiments conducted by the National Institute of Fisheries Science [20]. In these experiments, UPSs harvested in Wando, Korea, in April 2023, were subjected to blanching treatments (NBL: 15 °C for 0.5 min; BL: 70 °C for 3 min), rinsed with running tap water at ambient temperature for 1 min, dried in a convection dryer at 55 °C for 72 h, and ground to 1 mm particles. The processed material was incorporated at a 20% inclusion level into a basal feed mixture of wheat bran, corn, and rice bran (1:1:1 ratio) and subsequently ensiled in 1 kg silos under anaerobic conditions for 45 days.

Methane reduction was quantified following the methodological framework described by Adesogan et al. [33], employing a two-factor experimental design with blanching treatment (non-blanched vs. blanched) and supplementation levels (1.25%, 2.5%, and 5%) as fixed factors, with four replicates per treatment. Rumen fluid was collected from cannulated Hanwoo heifers fed a basal diet consisting of rice straw and commercial concentrate at an 8:2 ratio. The rumen fluid was pooled, strained through cheesecloth, and combined with an anaerobic buffer medium at a 1:2 (rumen fluid/buffer) ratio to prepare the inoculum. Incubation bottles (125 mL) containing 40 mL of the buffered rumen fluid and 0.5 g of the respective feed treatment were maintained at 39 °C for 48 h. At the end of incubation, total gas production and methane concentration were measured, and the relative reduction in methane emission compared to the control was calculated. The collected gas samples were analyzed for CO_2_ and CH_4_ using a gas chromatograph (Agilent Technologies HP 5890, Santa Clara, CA, USA), which was fitted with a thermal conductivity detector and a Carboxen 1006 PLOT capillary column measuring 30 mm × 0.53 mm (Supelco, Bellefonte, PA, USA).

In addition to methane concentration, supplementary fermentation parameters were measured to provide a more comprehensive assessment of ruminal fermentation balance. Specifically, pH was monitored as an indicator of acidity, volatile fatty acids were analyzed to reflect carbohydrate fermentation, ammonia-N was evaluated as a proxy for protein degradation, and feed digestibility was determined to assess nutrient utilization. Consideration of these parameters enabled a more robust interpretation of methane reduction outcomes within the broader context of ruminal fermentation dynamics.

For the economic valuation of methane abatement, baseline methane emissions per head were estimated using sex- and age-specific enteric fermentation coefficients provided by the Rural Development Administration of Korea [34]. To avoid confounding factors associated with reproduction, the analysis was restricted to Hanwoo steers. Applying the average fattening period of 31.1 months from 2022 to 2024, the baseline methane emission was calculated at 137.2 kg CH_4_ per head. Table 1 summarizes the emission coefficients by age group—39 kg CH_4_/head/year for calves under one year, 59 kg for yearlings, and 66 kg for cattle older than two years—which collectively serve as the basis for this baseline estimate. Methane emissions were subsequently converted into CO_2_ equivalents using the global warming potential (GWP) factor of 27.2 for non-fossil methane, as reported by the IPCC (2023) [35]. On this basis, the per-head economic benefit of UPS utilization can be expressed as detailed below.

(7) $$\begin{array}{c} \mathrm{Methane}\mathrm{abatement}\mathrm{benefit}\left(\frac{KRW}{head}\right)=\mathrm{Methane}\mathrm{Emission}\left(\frac{kgCH_{4}}{head}\right)\times \mathrm{Reduction}\mathrm{rate}\left(\%\right)\;\\ \times \mathrm{GWP}\left(\frac{27.2kgCO_{2}}{kgCH_{4}}\right)\times \mathrm{Carbon}\mathrm{price}\left(\frac{KRW}{kgCO_{2}}\right) \end{array}$$

## 3. Empirical Results

According to the National Institute of Fisheries Science [20], when basal feed was supplemented with 20% UPSs, the effective inclusion levels of pure UPSs corresponded to 0.25%, 0.5%, and 1.0%. Since the economic effects vary depending on the inclusion ratio, three scenarios were analyzed for comparison.

### 3.1. Incremental Processing Cost of UPS Utilization

For Hanwoo steers, the additional feed-processing cost per head at the 0.25% inclusion level amounted to KRW 25,714, comprising KRW 12,194 for collection and transportation and KRW 13,520 for drying and processing. These costs scaled proportionally with UPS usage; they doubled at the 0.5% level (51,429 KRW/head) and quadrupled at the 1.0% level (102,857 KRW/head).

### 3.2. Cost Savings from Disposal Avoidance and Import Substitution

At the 0.25% inclusion level, the use of UPSs in feed generated savings of KRW 9232 per head from reduced marine disposal costs and KRW 6839 per head from the substitution of imported feed ingredients, yielding a total of KRW 16,071 per head. As shown in Table 2, these savings also scaled proportionally with the inclusion rate, reaching 32,142 KRW/head at 0.5% and 64,284 KRW/head at 1.0%. Since the cost savings remained lower than the incremental expenditures, the adoption of UPSs in cattle feed would not be economically viable in the absence of additional benefits from methane emission reduction.

### 3.3. Benefits from Methane Emission Reduction

The benefits of methane abatement from UPS utilization were quantified based on the reduction rates reported by the National Institute of Fisheries Science [20]. Across all supplementation levels, desalted UPSs demonstrated superior efficacy compared with untreated samples. When incorporated into cattle feed at inclusion levels of 0.25%, 0.5%, and 1.0%, desalted UPSs significantly reduced methane emissions by 21.7%, 28.1%, and 53.2%, respectively, at the 5% significance level. While the difference between the 0.25% and 0.5% inclusion levels was relatively modest, the effect at the 1.0% level was substantially larger.

To monetize these reductions, Korea’s carbon allowance price was applied. Although allowance prices fluctuated over time, the highest observed value—KRW 40,000 per ton of CO_2_—was adopted in this analysis. Based on this assumption, the estimated per-head benefits of methane abatement were KRW 32,400, KRW 41,956, and KRW 79,433 at the 0.25%, 0.5%, and 1.0% inclusion levels, respectively. The results are summarized in Table 3.

### 3.4. Overall Economic Effects of UPS Utilization

The overall economic effects of UPS incorporation were derived by aggregating incremental processing costs, avoided disposal and substitution savings, and the monetized benefits of methane abatement. When the latter were internalized through Korea’s Emissions Trading Scheme, the estimated net cost savings per head amounted to KRW 22,757, KRW 22,669, and KRW 40,859 at inclusion levels of 0.25%, 0.5%, and 1.0%, respectively. Net savings at the 0.25% and 0.5% inclusion levels remained nearly identical, as the increase in processing costs was largely offset by comparable gains from disposal avoidance, import substitution, and methane abatement. At the 1.0% level, however, the substantially larger methane abatement benefits led to markedly higher overall savings. Relative to the average production cost of Hanwoo steers from 2022 to 2024 (8940,080 KRW/head) [26], the cost reduction ratios corresponded to 0.255%, 0.254%, and 0.457%. Although the marginal improvement between the 0.25% and 0.5% inclusion levels was limited, the effect observed at 1.0% was nearly double, highlighting the disproportionate gains at higher supplementation rates. These results are summarized in Table 4, which reports the incremental costs, cost savings, methane abatement benefits, and resulting net savings across the three inclusion levels. Detailed calculation procedures for Table 4 are provided in Supplementary Table S1.

At the sectoral level, scaling these values by the average annual number of Hanwoo steers from 2022 to 2024 (468,105 head) [26] yields changes in consumer surplus, producer surplus, and total social surplus. At inclusion levels of 0.25%, 0.5%, and 1.0%, consumer surplus increased by KRW 3459 million, KRW 3446 million, and KRW 6208 million, respectively. Producer surplus increased more substantially—KRW 7188 million, KRW 7160 million, and KRW 12,900 million—exceeding consumer gains by more than twofold. The corresponding total welfare improvements amounted to KRW 10,647 million, KRW 10,606 million, and KRW 19,108 million, with the 1.0% inclusion scenario demonstrating the largest welfare enhancement. These results are summarized in Table 5, which presents the estimated changes in consumer surplus, producer surplus, and total welfare across the three supplementation scenarios. Detailed calculations procedures for Table 5 are provided in Supplementary Table S2.

### 3.5. Sensitivity Analysis of Carbon Allowance Prices

In the baseline analysis, Korea’s carbon allowance price was fixed at KRW 40,000 per ton of CO_2_, representing the historically highest level. However, since 2024, the price declined sharply to approximately KRW 10,000 per ton [36]. To examine how economic outcomes vary with alternative price levels, additional simulations were conducted at 10,000 KRW/CO_2_ ton and 20,000 KRW/CO_2_ ton.

When the carbon allowance price was set at 10,000 KRW/CO_2_ ton, welfare effects were negative across all inclusion levels, amounting to KRW −722 million, KRW −4119 million, and KRW −8765 million at 0.25%, 0.5%, and 1.0%, respectively. The magnitude of welfare losses increased with higher supplementation rates, as incremental processing costs outweighed the benefits of methane abatement. By contrast, at 20,000 KRW/CO_2_ ton, welfare gains of KRW 3069 million, KRW 792 million, and KRW 535 million were observed at 0.25%, 0.5%, and 1.0% inclusion levels, respectively, indicating that total welfare increased but was larger at lower inclusion rates. This pattern reflects the fact that methane abatement benefits rise with supplementation, but other costs grow even more rapidly. Compared with the 40,000 KRW/CO_2_ ton scenario, the welfare gains at KRW 20,000 were modest in scale and displayed an opposite relationship with inclusion rates, underscoring the strong sensitivity of economic viability to carbon price levels. These results are presented in Table 6, which summarizes the economic effects of UPS utilization under alternative carbon allowance price scenarios.

## 4. Discussion

This study assesses the economic feasibility of utilizing UPSs, major by-products of seaweed aquaculture in Korea, as feed ingredients for cattle by estimating changes in social surplus, measured through consumer and producer surplus. The analysis shows that the additional costs of collection, transport, and processing outweigh the combined savings from avoided disposal and feed substitution. Accordingly, UPS utilization cannot be economically justified solely on these grounds. These results diverge from those of Kim et al. [21], who reported sufficient economic feasibility of seaweed by-products as feed based on feed substitution and methane abatement benefits. Their analysis, however, relied on the restrictive assumption of a static market equilibrium, which did not account for supply adjustments triggered by changes in production costs. By incorporating the most recent scientific evidence together with equilibrium adjustments, the present study improves upon earlier work and provides a more realistic assessment of the economic potential of UPS-based feed.

The realization of economic viability hinges on the internalization of methane abatement benefits as tangible reductions in production costs. This imperative calls for robust institutional mechanisms, notably greenhouse gas offset programs. With Korea’s Emissions Trading Scheme already in operation and multiple offset methodologies established for the agriculture, forestry, and fisheries sectors [37,38], UPS-based feed could be seamlessly embedded within these frameworks upon commercialization.

The viability of UPS-based feed is critically contingent upon the level of carbon allowance prices. When the price falls to KRW 10,000 per ton of CO_2_, as observed after 2024, even the 1% inclusion scenario—despite delivering the highest methane reduction rate—yields a decline in total welfare. Given that Korea’s allowance prices remain substantially below those prevailing in the European Union and other advanced markets [39], regulatory interventions to elevate carbon prices in alignment with Nationally Determined Contribution (NDC) targets will be indispensable [40]. Yet the volatility of carbon markets implies that reliance on this mechanism alone cannot provide a stable foundation for sustaining the UPS feed industry. Complementary measures, including direct payments for low-carbon livestock production and targeted feed subsidies, are therefore essential to ensure long-term viability.

Another salient finding is the distributional asymmetry of welfare gains: producer surplus expands by more than twice the magnitude of consumer surplus. This asymmetry suggests that policy instruments should be oriented primarily toward incentivizing producers. Mechanisms such as offset projects and producer-focused subsidies could play a decisive role in alleviating production costs and accelerating the adoption of UPS-based feed within the livestock sector.

Beyond immediate economic considerations, UPS utilization carries significant strategic value for enhancing feed security. Given Korea’s acute dependence on imported feed ingredients, even partial substitution with domestically available alternatives such as UPSs could bolster resilience, improve systemic robustness, and strengthen the long-term sustainability of livestock production systems. This broader societal dividend extends well beyond the direct cost savings quantified in this study and warrants more comprehensive investigation in future research.

Notwithstanding these contributions, several limitations should be acknowledged. In the absence of official statistics or commercialized cost data specific to UPSs, processing costs were estimated using proxy values derived from food waste management, which may not fully capture the distinct logistical characteristics of UPSs such as their high moisture content, seasonal variability, and geographic concentration. The analysis also focused primarily on economic costs and excluded life-cycle environmental impacts such as greenhouse gas emissions from drying, energy consumption, or water use. A comprehensive life-cycle assessment would provide a more complete appraisal of the environmental sustainability of UPS-based feed but remains a task for future research. Another limitation is that the methane reduction effects reported here were derived exclusively from in vitro fermentation experiments under a single feed condition using pooled rumen fluid, which limits the generalizability of the results. Further validation through in vivo feeding trials under diverse dietary conditions will be essential to confirm these findings. Finally, this study did not examine the effects of UPS supplementation on feed intake or growth performance, which are critical for determining its nutritional and economic value. Preliminary evidence from Korea suggests potential improvements in carcass grading and nutrient composition, supported by a registered patent (KR100401486B1) [41], but rigorous empirical validation is required before these findings can be generalized.

In addition to these analytical and methodological limitations, the safety aspects of UPSs as feed ingredients also warrant careful consideration. The feed safety of *Undaria pinnatifida* sporophylls (UPSs) was generally supported under the conditions tested. In this study, a blanching process (70 °C, 3 min) was applied to reduce mineral and heavy-metal contents, and safety was evaluated with reference to EU Directive 2002/32/EC [42] and international feed safety standards. According to internal analyses conducted as part of an ongoing patent and publication process, total arsenic in blanched UPSs remained below the maximum level for seaweed-derived feed materials specified by EU Directive 2002/32/EC (40 mg kg^−1^, 12% moisture basis). Although iodine was not directly analyzed in this study, previous work has shown that water-based processing methods such as soaking, blanching, and boiling can substantially reduce iodine concentrations in brown seaweeds through the leaching of water-soluble iodine species into the processing water [43]. Given the low inclusion level (<1.0% of dietary dry matter) and the blanching treatment applied, the risk of excessive iodine intake, long-term accumulation, or toxicity is considered minimal. Previous studies using brown seaweed by-products or calcareous red-algae-derived buffers also reported no adverse effects on milk yield, composition, or blood biochemical indices [2,11], although minor endocrine responses have been noted. To further substantiate feed safety, targeted quantification of iodine and long-term in vivo validation studies will be required in future research phases to verify the absence of residue accumulation or physiological side effects.

Empirical evidence further confirms that a 1% inclusion rate achieved the most substantial reduction in methane emissions (53.2%), more than double that of the 0.25% scenario, and generated the largest welfare gains under a high carbon price assumption (KRW 19,108 million at KRW 40,000 per ton of CO_2_). These findings indicate that a 1% inclusion level may optimize both environmental and economic outcomes, although further empirical validation is required before definitive policy conclusions can be drawn. In this regard, ongoing trials by the National Institute of Fisheries Science [19,20] are administering controlled feeding experiments with cattle to verify the effectiveness of UPS-based feed. For analytical convenience, the present study was limited to Hanwoo steers. Future investigations should expand the scope to incorporate cows and dairy cattle and evaluate possible implications for product quality, including both beef and milk.

Taken together, the evidence demonstrates that the economic viability of UPS-based cattle feed depends critically on the internalization of environmental benefits, the stabilization of carbon markets, and the implementation of producer-centered policy instruments. Supported by robust institutional frameworks and complemented by carefully calibrated policy measures, UPS utilization could make a substantive contribution both to the sustainable valorization of seaweed by-products and to the decarbonization of Korea’s livestock sector.

## 5. Conclusions

This study assessed the economic and environmental viability of utilizing UPSs, major by-products of seaweed aquaculture, as cattle feed in South Korea. The results showed that without environmental valuation, the additional costs of collection, transport, and processing exceeded the savings from disposal avoidance and import substitution, limiting standalone feasibility. When methane abatement benefits were monetized under Korea’s Emissions Trading Scheme, however, significant net benefits emerged, especially at higher inclusion levels, with producer surplus gains consistently exceeding consumer gains.

These findings demonstrate that the viability of UPS-based feed critically depends on effective institutional mechanisms, notably carbon pricing and producer-oriented support measures. Beyond direct cost savings, UPS utilization offers broader co-benefits for climate change mitigation and domestic feed security. By transforming a discarded by-product into a strategic resource, UPS feed can contribute to both sustainable aquaculture and livestock decarbonization, provided that robust policy frameworks are in place and further empirical validation is pursued.

## Figures and Tables

**Table 1:** Enteric methane emission coefficients for Hanwoo cattle by age group.

Age Group	Average Age (Months)	Emission Coefficients (kgCH_4_/Head/Year)
Under 1 year	<12	39
1–2 years	12–24	59
Over 2 years	>24	66
Steers (total period)	31.1	137.2

**Table 2:** Incremental costs and cost savings by UPS inclusion level.

Category		UPS Inclusion Rate		
		0.25%	0.5%	1.0%
Incremental cost (KRW/head)	Collection and transport	12,194	24,388	48,776
	Drying and processing	13,520	27,041	54,082
	Subtotal	25,714	51,429	102,857
Cost savings (KRW/head)	Disposal avoidance	9232	18,465	36,929
	Substitution of imported feed	6839	13,677	27,354
	Subtotal	16,071	32,142	64,284

**Table 3:** Methane reduction rates and associated abatement benefits by UPS inclusion level.

Category	UPS Inclusion Rate		
	0.25%	0.5%	1.0%
Methane reduction rate (%)	21.7%	28.1%	53.2%
Methane abatement benefits (KRW/head)	32,400	41,956	79,433

Note: Carbon price is assumed at 40,000 KRW/CO_2_ ton.

**Table 4:** Net cost savings and cost reduction by UPS inclusion level (KRW/head).

Category	UPS Inclusion Rate		
	0.25%	0.5%	1.0%
Incremental costs (A)	25,714	51,429	102,857
Cost savings (B)	16,071	32,142	64,284
Methane abatement benefits (C)	32,400	41,956	79,433
Net cost savings (−A+B+C)	22,757	22,669	40,859
Cost reduction ratio (%)	0.255	0.254	0.457

**Table 5:** Economic effects of UPS utilization by inclusion level (million KRW).

Category	UPS Inclusion Rate		
	0.25%	0.5%	1.0%
Consumer surplus change (A)	3459	3446	6208
Producer surplus change (B)	7188	7160	12,900
Total welfare change (A+B)	10,647	10,606	19,108

**Table 6:** Economic effects under alternative carbon allowance price scenarios.

Carbon Price	Category	UPS Inclusion Rate		
		0.25%	0.5%	1.0%
10,000 KRW/CO_2_ ton	Methane abatement benefits	8100	10,489	19,858
	Net cost savings	−1543	−8798	−18,715
	Cost reduction ratio (%)	−0.017	−0.098	−0.209
	Total welfare change (million KRW)	−722	−4119	−8765
20,000 KRW/CO_2_ ton	Methane abatement benefits	16,200	20,978	39,716
	Net cost savings	6557	1691	1143
	Cost reduction ratio (%)	0.073	0.019	0.013
	Total welfare change (million KRW)	3069	792	535

## Data Availability

The original data presented in this study are openly available in references [20,23,24,25,26,27,28,30,31,32,34,35,36].

## Supplementary Materials

The following supporting information can be downloaded at: https://www.mdpi.com/article/10.3390/ani15223260/s1, Table S1: Net cost savings and cost reduction by UPS inclusion level, Table S2: Economic effects of UPS utilization by inclusion level.

## Abbreviations

The following abbreviations are used in this manuscript:

UPSs	*Undaria pinnatifida* sporophylls
